# Ultrasound techniques for the detection of developmental dysplasia of the hip: a systematic review and meta-analysis

**DOI:** 10.1590/1516-3180.2021.0852.13062022

**Published:** 2022-08-29

**Authors:** Marcio Luís Duarte, Giovanna Galvão Braga Motta, Natasha Vogel Majewski Rodrigues, Alessandra Rodrigues Silva Chiovatto, Eduardo Davino Chiovatto, Wagner Iared

**Affiliations:** I MD, MSc. Musculoskeletal Radiologist, WEBIMAGEM Telerradiologia, São Paulo (SP), Brazil. Doctoral student in Evidence-based Health Program, Universidade Federal de São Paulo (UNIFESP), São Paulo (SP), Brazil.; Universidade Federal de São Paulo, São Paulo, SP, Brazil; II MD, MSc. Radiologist, Centro de Aperfeiçoamento e Pesquisa em Ultrassonografia Prof. Dr. Giovanni Guido Cerri (DASA), Ultrasonography, São Paulo, Brazil. Doctoral Student in Evidence-based Health Program, Universidade Federal de São Paulo (UNIFESP), São Paulo (SP), Brazil.; Universidade Federal de São Paulo, São Paulo, SP, Brazil; III MD, MSc. Pediatric Orthopedist, Hospital do Servidor Público Municipal, Pediatric orthopedics, São Paulo (SP), Brazil.; IV MD. Radiologist, Centro de Aperfeiçoamento e Pesquisa em Ultrassonografia Prof. Dr. Giovanni Guido Cerri (DASA), Ultrasonography, São Paulo (SP), Brazil. Brazil.; V MD. Radiologist, Centro de Aperfeiçoamento e Pesquisa em Ultrassonografia Prof. Dr. Giovanni Guido Cerri (DASA), Ultrasonography, São Paulo (SP), Brazil. Brazil.; VI MD, PhD. Radiologist and Supervisor Professor, Evidence-Based Health Postgraduate Program, Universidade Federal de São Paulo (UNIFESP), São Paulo (SP), Brazil.

**Keywords:** Hip dislocation, congenital, Ultrasonography, Hip joint, Infant, newborn, Developmental dysplasia of the hip, Ultrasound, Hip dysplasia, DDH, Infantile hip dysplasia

## Abstract

**BACKGROUND:**

Developmental dysplasia of the hip (DDH) encompasses a broad spectrum of hip pathologies, including femoral or acetabular dysplasia, hip instability, or both. According to the medical literature, ultrasonography is the most reliable diagnostic method for DDH. Several techniques for the assessment of hips in newborns and infants, using ultrasonography, have been described.

**OBJECTIVE:**

To compare the accuracy of the Graf technique and other diagnostic techniques for DDH.

**DESIGN AND SETTING:**

A systematic review of studies that analyzed ultrasound techniques for the diagnosis of DDH within an evidence-based health program of a federal university in São Paulo (SP), Brazil.

**METHODS:**

A systematic search of relevant literature was conducted in the PubMed, EMBASE, Cochrane Library, CINAHL, and LILACS databases for articles published up to May 5, 2020, relating to studies evaluating the diagnostic accuracy of different ultrasound techniques for diagnosing DDH. The QUADAS 2 tool was used for methodological quality evaluation.

**RESULTS:**

All hips were analyzed using the Graf method as a reference standard. The Morin technique had the highest rate of sensitivity, at 81.12–89.47%. The Suzuki and Stress tests showed 100% specificity. The Harcke technique showed a sensibility of 18.21% and specificity of 99.32%.

**CONCLUSION:**

All the techniques demonstrated at least one rate (sensibility and specificity) lower than 90.00% when compared to the Graf method. The Morin technique, as evaluated in this systematic review, is recommended after the Graf method because it has the highest sensitivity, especially with the three-pattern classification of 89.47%.

**REGISTRATION NUMBER:**

Identifier: CRD42020189686 at the International Prospective Register of Systematic Reviews (identifier: CRD42020189686).

## INTRODUCTION

Developmental dysplasia of the hip (DDH) encompasses a broad spectrum of hip pathologies, including femoral dysplasia, acetabular dysplasia, hip instability, and any combination of these, as well as the subluxation or dislocation of the femoral head.^1–5^ Although the exact cause of DDH remains unknown,^5^ it is the most common congenital abnormality of the musculoskeletal system,^4,6^ with an incidence of 1.6–28.5 cases per 1,000 live births and a prevalence of 0.15–4.0%.^5,7–11^ Of individuals in whom congenital dislocation of the hip is not treated, up to 94% of individuals will develop moderate or severe osteoarthritis in the second decade of life.^8^

Although DDH was first described more than two millennia ago, there is still some controversy regarding the etiology, diagnosis, and methods of treatment.^12,13^ Early diagnoses became more meaningful after it was discovered that hip dysplasia was not only genetic, but also developmental.^8^ Studies on the diagnosis, monitoring, and treatment of DDH have produced results that are controversial or contradictory.^12^ Those discrepancies could be attributable to a variation in the physiological development of the hip being misinterpreted as a pathological process, to differences in the terminology employed by radiologists and clinicians, or to differences in the physical examination and hip ultrasound standards.^12^

An early diagnosis of DDH aids in the prognosis and success of treatment, especially non-surgical treatment.^7,14–16^ Approximately 10% of all hip arthroplasty procedures in adults are performed to correct disorders that arise in childhood, primarily DDH.^15^ A diagnostic delay of three months or more increases the probability of surgery being needed to correct the problem.^4^ A diagnosis of DDH is the indication for hip arthroplasty in up to 9% of patients under 65 years of age and in 25% in those under 40 years of age who develop premature arthrosis.^4,8,11,17,18^

In cases in which DDH is treated inappropriately, the main complication is avascular necrosis of the femoral head.^5,11,19^ In such cases, the diagnostic method of choice is magnetic resonance imaging.^20^ The risk factors for DDH include the following:^1,19^

- Family history
- Female sex (4–6 times higher risk)
- First-born status
- Low birth weight (< 2,500 g)
- Oligohydramnios
- Breech position *in utero*
- Prematurity (< 37 weeks of gestation)
- Twinning
- The practice of swaddling (wrapping the newborn tightly in cloth), which keeps the hips in an extended, adducted position that can create an abnormal relationship between the head of the femur and acetabulum

Despite being operator-dependent, ultrasonography is considered as the most reliable method for the diagnosis of DDH in the neonatal period.^12,17,21,22^ It is a noninvasive method that does not involve the use of radiation, and is portable and easy to use. However, a physician must perform more than 100 ultrasound examinations to be considered as qualified.^13,15,23–26^ Ultrasonography of the hip detects 52% more pathological hips than the Ortolani and Barlow tests.^14^ In addition, ultrasonography makes it possible to perform a dynamic study and the Ortolani and Barlow maneuvers simultaneously.^14,15^ Various techniques have been described for the ultrasound assessment of hips in newborns and infants, although there is no consensus as to which technique is the best.^27,28^

## OBJECTIVE

The objective of this study was to determine the detection rates and accuracy of different two-dimensional ultrasound techniques for the diagnosis of DDH using the Graf method as a reference. To this end, we conducted a systematic review of the literature on this topic.

## METHODS

### Study model

The study design followed the model outlined in the Cochrane Handbook for Systematic Reviews of Diagnostic Test Accuracy, version 5.1.^29^ The review was registered with the International Prospective Register of Systematic Reviews (identifier: CRD42020189686).

### Inclusion criteria

This review was performed in accordance with the Preferred Reporting Items for Systematic Reviews and Meta-Analyses statement.^30^ We included comparative studies on the diagnostic accuracy of the Graf technique and at least one other technique for diagnosing DDH in the first year of life, among patients with or without risk factors for the condition. The other techniques included the Finnbogason, Harcke, Morin, Rosendahl, stress test, Suzuki, Terjesen, and Tréguier techniques. We did not impose any restrictions with respect to the patient origin, article language, sample size, or publication status of the studies.

### Patients

Among the selected studies, all patients were of age ≤ 12 months. The study sample included infants who underwent ultrasound for routine screening or were considered to be at a high risk for DDH.

### Study selection and data extraction

The selected studies were those potentially eligible for inclusion in terms of the relevance of the abstracts or full texts. Two authors, working independently, determined their eligibility. Disagreements were resolved through a consensus. Data extraction was performed using a standardized form. The selection process was carried out on the Rayyan platform.^31^ In case of missing data, we contacted the authors of the study by e-mail.

### Evaluation of the methodological quality

For all the eligible studies, we employed the Quality Assessment of Diagnostic Accuracy Studies 2 tool,^32^ which focuses on the evaluation of bias and accuracy. All analyses were performed and all diagrams were created using the Review Manager program (version 5.3, RevMan; Cochrane Collaboration, Oxford, United Kingdom). The Review Manager program was used to calculate the sensitivity and specificity, as well as the corresponding 95% confidence intervals (CIs), for the previously mentioned criteria.

### Search strategies

We performed a thorough systematic search for original articles in the following databases (from inception to May 5, 2020): PubMed, Excerpta Medica, Cochrane Library, Cumulative Index to Nursing and Allied Health Literature, and Latin-American and Caribbean Health Sciences Literature. We used the National Library of Medicine Medical Subject Headings “Hip Dislocation, Congenital” and “Ultrasonography,” together with the term “Graf.” Additionally, we performed manual searches of the reference lists of the included studies and evaluated the main reviews of the subject. Appendix 1 provides the full search strategy.

### Evaluated techniques of ultrasonography

#### Graf

The Graf method consists of the evaluation of a conventional coronal view with the patient in the lateral position, providing qualitative and quantitative assessments of the hip.^17,33–35^ The Graf method classifies the degree of coverage of the bony acetabular roof (alpha angle) and cartilaginous acetabular roof (beta angle). For the meta-analysis, we considered that following the guidance provided by Graf,^36^ a type IIA-hip was an indication for treatment.

#### Morin

In the Morin technique,^37^ a coronal image of the flexed hip was evaluated to estimate the percentage of the femoral head that was medial to the lateral iliac margin (the “iliac line,” resembling the Graf “baseline”) and consequently covered by the bony acetabulum. The studies analyzed used different classifications of normal test results. Therefore, each study was evaluated individually.

#### Suzuki

In the Suzuki technique,^14,38^ the hips of the patient were maintained in abduction (in flexion or extension) and a long linear probe was positioned transversely over the lower pelvis in the region of the pubic bones. The purpose was to delineate the location of the femoral head. In the meta-analysis, we considered that a slight dislocation should not be classified as a normal test result and was an indication for treatment.

#### Terjesen

In the Terjesen technique,^27,28,39^ a coronal profile image was evaluated with the hip lightly flexed, and a line was traced parallel to the long axis of the ultrasound probe. The iliac bone should always be examined as a straight line parallel to the edge of the coronal mid-acetabular image. The analyzed studies used different classifications of normal test results. Therefore, we individually evaluated each classification.

#### Tréguier

Tréguier et al.^40^ defined the pubofemoral distance (PFD) as the distance between the most medial aspect of the femoral head and the most lateral aspect of the pubis. The Tréguier technique involved the measurement of the pubofemoral distance (PFD) in the coronal plane, which includes the largest circumference of the femoral head and the most lateral aspect of the pubis.

#### Harcke

In the Harcke technique,^13,14,17^ the patient was placed in the supine position, the hip was maneuvered through the neutral and flexed positions with and without the aid of stress (Barlow maneuver), and the lateral transverse and coronal aspects were evaluated. The main target was the femoral head at rest and during the stress examination.

#### Finnbogason

In the Finnbogason technique,^41,42^ the patient was placed in the supine position and the ultrasound probe was positioned anterior and parallel to the longitudinal axis of the femoral neck. This produced an oblique sagittal image of the hip, including the anterior acetabular rim as well as the femoral head and neck. The probe was placed in a holder, which allowed the physician to have both hands free. The physician employed downward pressure, with the target hip in the flexion and mid-abduction positions (Barlow maneuver) with one hand while using the other hand to keep the patient in the correct position. In the meta-analysis, an unstable hip was classified as abnormal.

#### Stress test

For the ultrasound stress test,^43^ the patient was placed in the lateral position, and a dynamic stress test was performed in the coronal plane, with the hip in flexion. For the meta-analysis, a lax hip was classified as abnormal.

#### Rosendahl

In the Rosendahl technique,^44^ the patient was placed in the lateral position, the ultrasound probe was positioned laterally, and the physician performed a stress test (adjusted Barlow maneuver) with one hand while using the other hand to maintain the ultrasound probe in the correct position. In this meta-analysis, an elastic hip was classified as abnormal.

## RESULTS

### Selected studies

**Figure 1:**
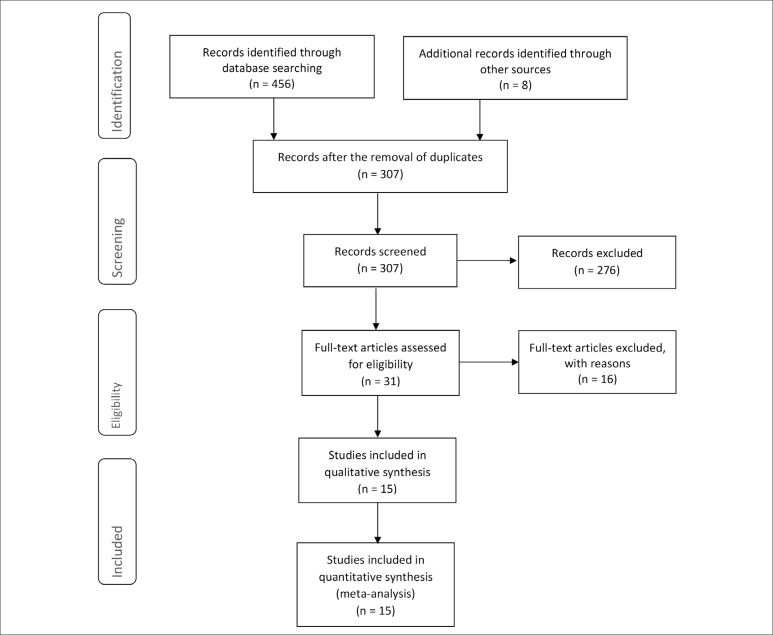
Preferred Reporting Items for Systematic Reviews and Meta-Analyses flow diagram.

We conducted a systematic review of 494 studies. At the end of the selection process, 15 studies were deemed to meet the inclusion criteria and present acceptable quality, as determined using the Quality Assessment of Diagnostic Accuracy Studies 2 tool. Therefore, all 15 studies were included in the systematic review (Figure 1), as well as in the meta-analysis.

### Analysis on the studies

**Figure 2:**
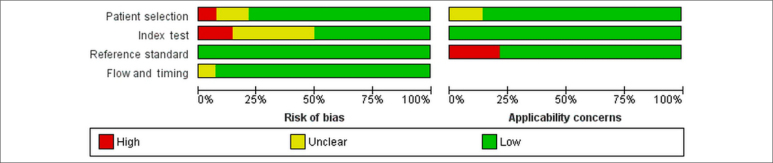
Risk of bias and applicability concerns, as determined with the Quality Assessment of Diagnostic Accuracy Studies 2 tool.

**Figure 3:**
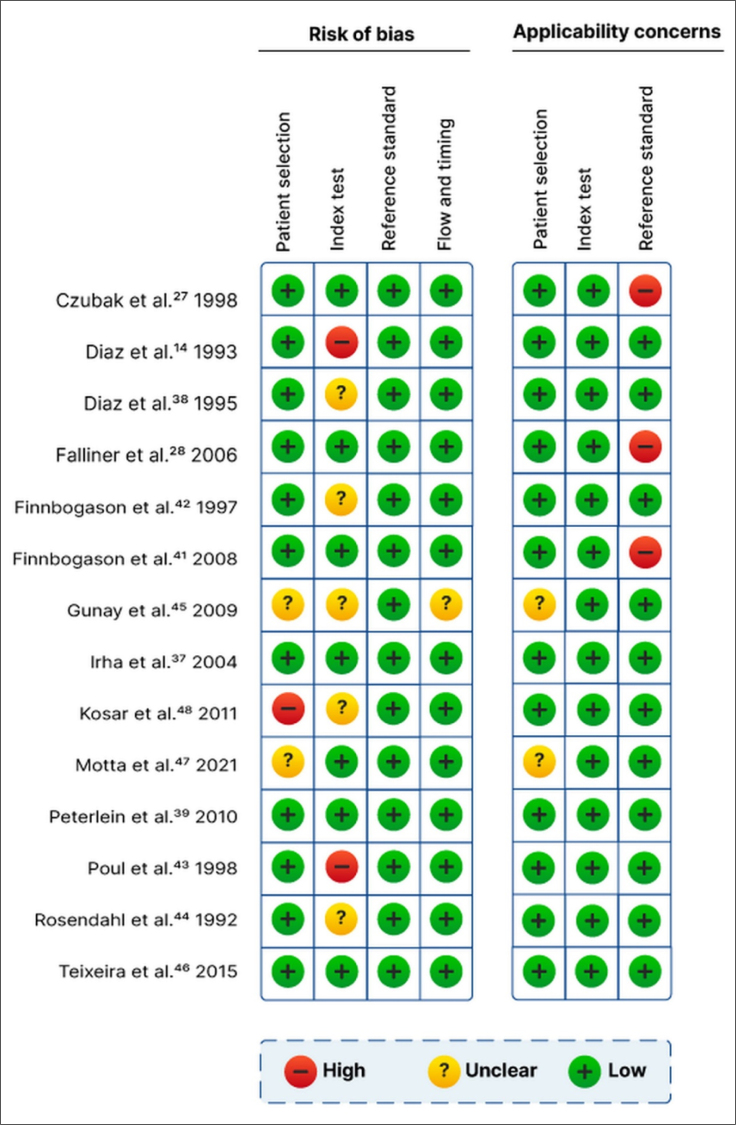
Summary risk of bias and applicability concerns, as determined with the Quality Assessment of Diagnostic Accuracy Studies 2 tool.

In one study, there was a concern of bias in patient selection because the study sample included only male patients. Two other studies did not describe the patient-selection process. In two studies, the comparative technique was performed after the results of the Graf method were known, and in five studies, the order of application of the methodologies was not noted. In three studies, there were concerns regarding the application of the Graf method because only the alpha angle was evaluated. All the patients were younger than 12 months of age. In most studies, the Graf method and comparative technique were performed on the same day (Figures 2 and 3). Overall, 15 studies evaluated 16,736 hips. The Graf method was compared with the Morin technique in two studies,^37,45^ with the Suzuki technique in two studies,^14,38^ with the Terjesen technique in three studies,^27,28,39^ with the Tréguier technique in two studies,^46,47^ with the Harcke technique in two studies,^14,48^ with the Finnbogason technique in two studies,^41,42^ with the stress test in one study,^43^ and with the Rosendahl technique in one study.^44^

As shown in Table 1A, Gunay et al.^45^ used the Morin technique to evaluate 2,074 hips, dividing the findings into two categories by the proportion of acetabular coverage of the femoral head: ≥ 51% (mature hip) and < 51% (immature hip). The authors found that the Morin technique had a sensitivity of 81.12% and specificity of 82.70% (P < 0.05), with an overall accuracy of 82.59%. In a study of 100 hips, Irha et al.37 also evaluated the Morin technique, dividing the findings into three categories according to the proportion of acetabular coverage of the femoral head: ≥ 58% (normal hip), 33.58% (borderline pathological hip), and < 33% (pathological hip). We considered hips with a coverage . 58% as normal when the three-category Morin technique was used because borderline cases could evolve to a pathological status. Irha et al.37 found a technique with a sensitivity of 89.47% and a specificity of 83.95% (P < 0.05), with an overall accuracy of 85.00% (Table 1B).

**Table 1A:** Summary of detection rates using the Morin technique with two categories

	Graf method		Morin technique	
	DDH/Hips evaluated	Detection rate	DDH/Hips evaluated	Detection rate
Gunay et al.,^45^ 2009	143/2074	6.89%	450/2074	21.69%

DDH = developmental dysplasia of the hip.

**Table 1B:** Summary of detection rates using the Morin technique with three categories

	Graf method		Morin technique	
	DDH/Hips evaluated	Detection rate	DDH/Hips evaluated	Detection rate
Irha et al.,^37^ 2004	19/100	19.00%	30/100	30.00%

DDH = developmental dysplasia of the hip.

Table 2 shows the detection rates for the Suzuki technique, which was analyzed in two studies.^14,38^ The technique was found to have a sensitivity of 39.36% and a specificity of 100.00% (P < 0.05), with an overall accuracy of 69.21%. The two studies evaluated a total of 1,166 hips.

**Table 2:** Summary of detection rates using the Suzuki technique

	Graf method		Suzuki technique	
	DDH/Hips evaluated	Detection rate	DDH/Hips evaluated	Detection rate
Diaz et al.,^14^ 1993	206/416	49.51%	79/416	18.99%
Diaz et al.,^38^ 1995	386/750	51.46%	154/750	20.53%
**Total**	**592/1,166**	**50.77%**	**233/1,166**	**19.98%**

DDH = developmental dysplasia of the hip.

Falliner et al.^28^ and Peterlein et al.^39^ compared the Graf method with the Terjesen technique (Table 3A), evaluating a collective total of 878 hips and dividing the findings into two categories according to the proportion of acetabular coverage of the femoral head, each with separate cutoff values for male and female patients: ≥ 47% and ≥ 44%, respectively (normal hip), and < 47% and < 44%, respectively (pathological hip). Collectively, the two studies showed that the technique had a sensitivity of 14.41% and a specificity of 99.74% (P < 0.05), with an overall accuracy of 88.30%. In a study involving 1,312 hips, Czubak et al.^27^ also evaluated the Terjesen technique (Table 3B), dividing the hips into four categories according to the proportion of femoral head coverage: ≥ 50% (normal hip), 49–40% (possible hip dysplasia), 39–10% (hip subluxation), and < 10% (hip dislocation). In this systematic review, findings of possible dysplasia, subluxation, and dislocation were considered to be indicative of an abnormal hip. In the Czubak et al.^27^ study, the technique was found to have a sensitivity of 39.39% and specificity of 93.47% (P < 0.05), with an overall accuracy of 75.99%.

**Table 3A:** Summary of detection rates using the Terjesen technique with two categories

	Graf method		Terjesen technique	
	DDH/Hips evaluated	Detection rate	DDH/Hips evaluated	Detection rate
Falliner et al.,^28^ 2006	86/464	18.53%	19/464	4.09%
Peterlein et al.,^39^ 2010	32/414	7.72%	00/414	00.00%
**Total**	**118/878**	**13.43%**	**19 / 878**	**2.16%**

DDH = developmental dysplasia of the hip.

**Table 3B:** Summary of detection rates using the Terjesen technique with four categories

	Graf method		Terjesen technique	
	DDH/Hips evaluated	Detection rate	DDH/Hips evaluated	Detection rate
Czubak et al.,^27^ 1998	424/1,312	32.31%	225/1,312	17.14%

DDH = developmental dysplasia of the hip.

Teixeira et al.^46^ used the Tréguier technique to evaluate 232 hips under four different conditions (Table 4A):

- A hip in flexion with a PFD of 3.3 millimeters (mm)—sensitivity of 76.19% and a specificity of 64.21% (P < 0.05), with an overall accuracy of 66.38%.
- A hip in flexion with a PFD of 4.9 mm—sensitivity of 59.52% and a specificity of 88.95% (P < 0.05), with an overall accuracy of 83.62%.
- A hip in the neutral position with a PFD of 4.0 mm—sensitivity of 50.00%, specificity of 93.68% (P < 0.05), and an overall accuracy of 85.78%.
- A hip in the neutral position with a PFD of 4.6 mm—sensitivity of 50.00%, specificity of 93.68% (P < 0.05), and an overall accuracy of 85.78%.

**Table 4A:** Summary of detection rates using the Tréguier technique according to Teixeira et al.,^46^ 2015

	Graf method		Tréguier technique	
	DDH/Hips evaluated	Detection rate	DDH/Hips evaluated	Detection rate
Flexion 3.3 mm	42/232	18.10%	100/232	43.10%
Flexion 4.9 mm	42/232	18.10%	46/232	19.82%
Neutral 4.0 mm	42/232	18.10%	33/232	14.22%
Neutral 4.6 mm	42/232	18.10%	33/232	14.22%

DDH = developmental dysplasia of the hip.

In a similar study, Motta et al.^47^ applied the Tréguier technique to 1,980 hips, all of which were evaluated with the hip in flexion and with a PFD of 3.0 mm (Table 4B). The authors found that the technique had a sensitivity of 63.55% and a specificity of 62.22% (P < 0.05), with an overall accuracy of 62.42%.

**Table 4B:** Summary of detection rates using the Tréguier technique according to Motta et al.,^47^ 2021

	Graf method		Tréguier technique	
	DDH/Hips evaluated	Detection rate	DDH/Hips evaluated	Detection rate
Flexion 3.0 mm	310/1,980	15.65%	828/1,980	41.81%

DDH = developmental dysplasia of the hip.

Diaz et al.^14^ and Koşar et al.^48^ both evaluated the Harcke technique (Table 5). Collectively, the two studies showed that the technique had a sensitivity of 18.21% and specificity of 99.32% (P < 0.05), with an overall accuracy of 84.47%. The two studies evaluated a collective total of 3,058 hips.

**Table 5:** Summary of detection rates using the Harcke technique

	Graf method		Harcke technique	
	DDH/Hips evaluated	Detection rate	DDH/Hips evaluated	Detection rate
Diaz et al., ^14^ 1993	206/416	49.51%	79/416	18.99%
Koşar et al., ^48^ 2011	354/2,642	13.39%	40/2,642	1.51%
**Total**	**560/3,058**	**18.31%**	**119/3,058**	**3.89%**

DDH = developmental dysplasia of the hip.

As detailed in Table 6, the Finnbogason technique was evaluated in two separate studies.^41,42^ Collectively, the two studies showed that the technique had a sensitivity of 39.48% and specificity of 96.83% (P < 0.05), with an overall accuracy of 83.73%. Two studies evaluated a collective total of 1,186 hips.

**Table 6:** Summary of detection rates using the Finnbogason technique

	Graf method		Finnbogason technique	
	DDH/Hips evaluated	Detection rate	DDH/Hips evaluated	Detection rate
Finnbogason et al.,^42^ 1997	20/114	17.54%	05/114	4.38%
Finnbogason et al.,^41^ 2008	251/1,072	23.41%	131/1,072	12.22%
**Total**	**271/1,186**	**22.84%**	**136/1,186**	**11.46%**

DDH = developmental dysplasia of the hip.

Poul et al.^43^ applied the stress test technique to the evaluation of 1,744 hips (Table 7). The authors found that the technique had a sensitivity and specificity of 39.48% and 96.83%, respectively (P < 0.05), with an overall accuracy of 97.94%.

**Table 7:** Summary of detection rates using the Stress test technique

	Graf method		Stress test technique	
	DDH/Hips evaluated	Detection rate	DDH/Hips evaluated	Detection rate
Poul et al.,^43^ 1998	39/1,744	2.23%	03/1,744	0.17%

DDH = developmental dysplasia of the hip.

Table 8 shows the results of a study analyzing the accuracy of the Rosendahl technique in a sample of 3,006 hips.^44^ This technique was found to have a sensitivity of 50.78% and specificity of 97.51% (P < 0.05), with an overall accuracy of 89.49%.

**Table 8:** Summary of detection rates using the Rosendahl technique

	Graf method		Rosendahl technique	
	DDH/Hips evaluated	Detection rate	DDH/Hips evaluated	Detection rate
Rosendahl et al.,^44^ 1992	416/3,006	13.83%	324/3,006	10.77%

DDH = developmental dysplasia of the hip.

**Figure 4:**
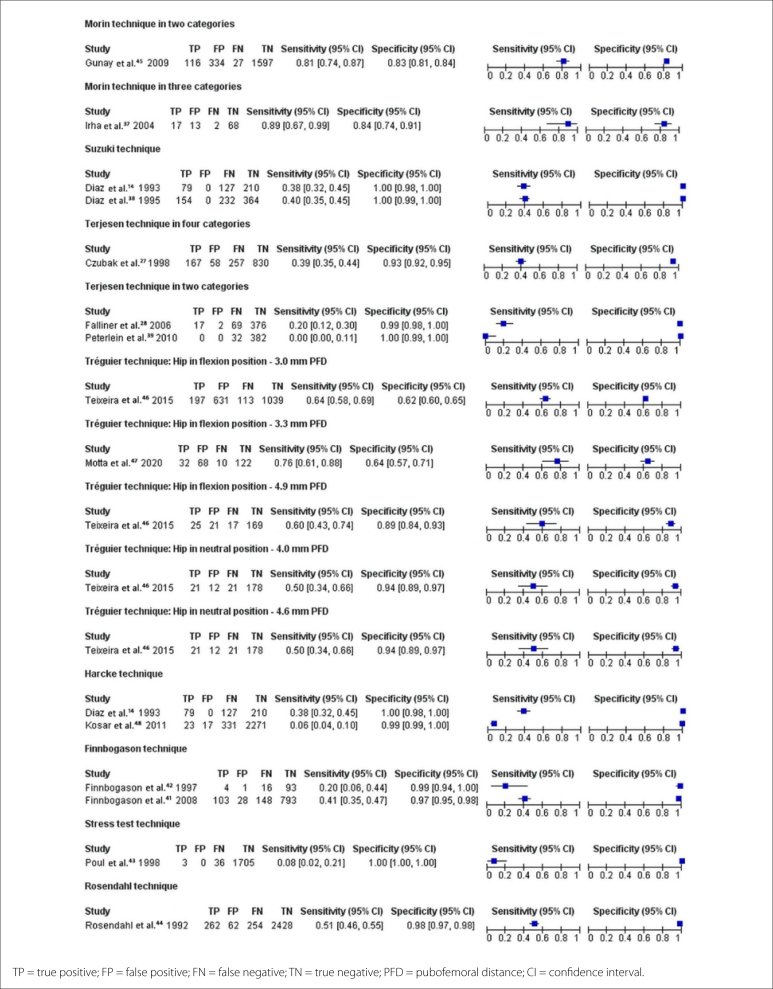
Forest plots of the sensitivity and specificity of the ultrasound techniques evaluated.

**Figure 5:**
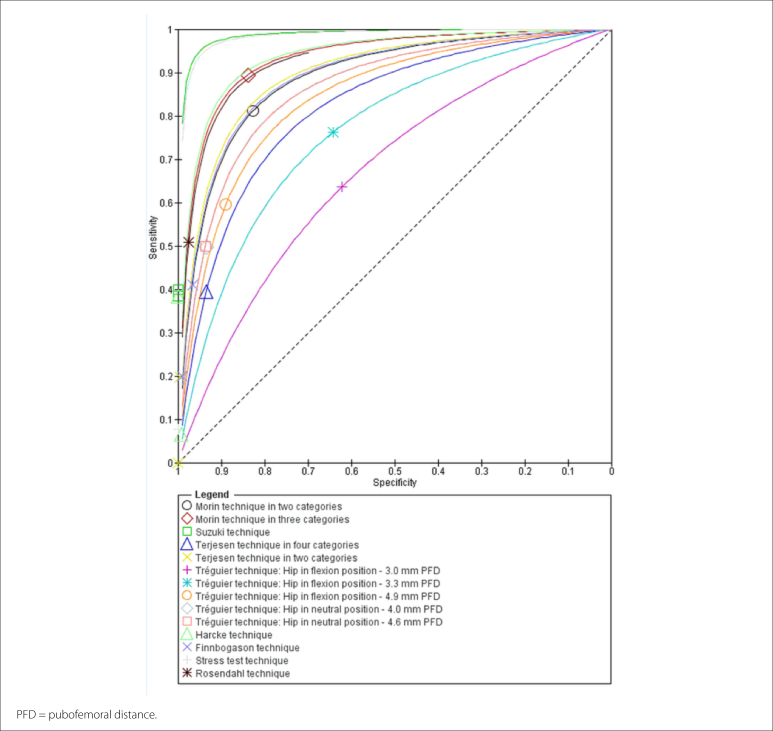
Summary receiver operating characteristic curves for the sensitivity and specificity of the ultrasound techniques evaluated.

The accuracy and DDH prevalence data for all 15 studies have been summarized in Table 9. The sensitivity and specificity data were also analyzed in forest plots (Figure 4), along with summary receiver operating characteristic curves (Figure 5).

**Table 9:** Summary of sensitivity, specificity, prevalence, and hips evaluated by all techniques

	Sensitivity	Specificity	Prevalence	Accuracy	Hips evaluated
Morin with two patterns	81.12%	82.70%	6.89%	82.59%	2,074
Morin with three patterns	89.47%	83.95%	19.00%	85.00%	100
Suzuki	39.36%	100.00%	50.77%	62.21%	1,166
Terjesen with two patterns	14.41%	99.74%	13.41%	88.30%	878
Terjesen with four patterns	39.39%	93.47%	32.32%	75.99%	1,312
Tréguier flexion 3.0 mm	63.55%	62.22%	15.66%	62.42%	1,980
Tréguier flexion 3.3 mm	76.19%	64.21%	18.10%	66.38%	232
Tréguier flexion 4.9 mm	59.52%	88.95%	18.10%	83.62%	232
Tréguier neutral 4.0 mm	50.00%	93.68%	18.10%	85.78%	232
Tréguier neutral 4.6 mm	50.00%	93.68%	18.10%	85.78%	232
Harcke	18.21%	99.32%	18.31%	84.47%	3,058
Finnbogason	39.48%	96.83%	22.85%	83.73%	1,186
Stress test	7.69%	100.00%	2.24%	97.94%	1,744
Rosendahl	50.78%	97.51%	17.17%	89.49%	3,006

## DISCUSSION

In the studies selected for review, the Graf method was used as a reference for the diagnosis of DDH. Among the other analyzed techniques, the Morin technique had the highest sensitivity (81.12%) when the proportion of acetabular coverage of the femoral head was divided into two categories and 89.47% when it was divided into three categories, whereas the specificity was 83.95% and 82.70%, respectively. In terms of the specificity, the techniques that showed the best performance were the Suzuki technique and stress test, both of which showed a specificity of 100.00%, compared with 99.74% for the two-category Terjesen technique, 99.32% for the Harcke technique, 96.83% for the Finnbogason technique, and 97.51% for the Rosendahl technique.

The technique that showed the stress test had the highest overall accuracy, which was found to be 97.94%, compared with that of the Graf method, although its sensitivity was low (7.69%). The Rosendahl technique provided the second-highest overall accuracy, which was 89.49%, compared with 88.30% for the two-category Terjesen technique, 85.00% for the three-category Morin technique, 83.73% for the Finnbogason technique, and 83.73% for the Harcke technique. The high accuracy of some of these techniques could be attributed to the low prevalence of DDH in the hips that were studied. The sensitivity of the Tréguier technique was highest (76.19%) when the hip was in flexion and the PFD was 3.3 mm, whereas the specificity and accuracy of the technique were highest (93.68% and 85.78%, respectively) when the hip was in the neutral position, regardless of the PFD. Techniques with the highest specificity were also those with the lowest sensitivity.

The most common methods of screening for DDH in newborns are serial physical examinations of the hip, using the Ortolani and Barlow maneuvers, and ultrasonography.^12,23^ The American Academy of Pediatrics recommends routine screening for DDH through clinical examination by qualified personnel.^49^ However, the physical examination does not safely diagnose dysplastic hips and may also fail to identify unstable or even dislocated hips.^12^ Regarding newborns who undergo universal ultrasound screening, 5–7% are treated for hip dysplasia, compared with only 2% of those who undergo clinical screening alone.^2^

The Graf method is the ultrasound technique preferred by most physicians and is most widely employed.^35^ Although relatively simple and reproducible, the Graf method requires that the image of the hip be acquired in a specific spatial plane and that anatomical landmarks are properly identified.^17^ Those requirements can be challenging, especially for less experienced examiners,^17^ and some studies have shown poor intraobserver and interobserver agreement.^50^ The main complaint related to the Graf method is that it requires considerable training. Nevertheless, the image recommended by the Graf method is the same as that recommended by other techniques. To perform a satisfactory examination, it is crucial to recognize eight anatomical markers of the hip, namely:^17^ acetabular bony rim, acetabular bony roof, acetabular hyaline cartilage, acetabular labrum, chondro-osseous junction, femoral head, hip joint capsule and synovial fold.

After the Graf method, which is considered to be the gold standard, the Morin technique is the second most recommended because it has the highest sensitivity, particularly when the three-category version of the technique is employed. Because the Morin technique is more easily performed and has a relatively high sensitivity and specificity, it could be used as a screening method in locations where there is no specialist with sufficient experience to perform the Graf method. If the Morin technique indicated a pathological hip, the patient was transferred to a referral center for evaluation using the Graf method. Owing to its low sensitivity, the Harcke technique is not recommended as a screening method. The Suzuki technique and the stress test both show high specificity and could therefore serve as complements to other techniques with high sensitivity, such as the Morin technique.

## CONCLUSION

The importance of this systematic review is to demonstrate the detection rates and accuracy of different techniques of ultrasound diagnosis of DDH using the Graf method as a reference. None of the techniques displayed a sensitivity greater than 90.00% compared to the Graf method; the most comparable is the Morin technique divided into three patterns of bony rim percentage coverage over the femoral head (89.47%). With respect to the specificity, only the Morin technique (82.00–84.00%) and three different measures with the flexioned hip in the Tréguier technique (62.00–89.00%) demonstrated a rate inferior to 90.00%. Regarding the accuracy, the stress test proposed by Poul showed a rate superior to 90.00% (97.94%), followed by the Rosendahl technique (89.49%) and the Terjesen technique, which was divided into two groups of femoral head cover (88.30%).

However, all techniques demonstrated at least one rate lower than 90.00% when compared to the Graf method. The Morin technique, as evaluated in this systematic review, is recommended after the Graf method because it has the highest sensitivity, especially with the three-pattern classification of 89.47%. The Morin technique is simpler than the Graf technique. With this advantage, the Morin technique can be used for screening in areas that do not have a professional with satisfactory expertise to perform the Graf method. In circumstances where the Morin technique defines an unhealthy hip, the patient is forwarded to a reference location for a specific test using the Graf method.

## Appendix

**Appendix 1:** Search strategy by database

Database	Search strategy
Cochrane Library	1: MeSH descriptor: [Hip Dislocation, Congenital] explode all trees #2: MeSH descriptor: [Ultrasonography] explode all trees #3: “Graf” #4: #1 AND #2 AND #3
MEDLINE	#1: “Hip Dislocation, Congenital”[MeSH] OR (Congenital Hip Dislocations) OR (Dislocations, Congenital Hip) OR (Hip Dislocations, Congenital) OR (Congenital Hip Dislocation) OR (Congenital Hip Displacement) OR (Congenital Hip Dysplasia) OR (Congenital Hip Dysplasias) OR (Dysplasias, Congenital Hip) OR (Hip Dysplasias, Congenital) OR (Hip, Dislocation Of, Congenital) OR (Dislocation, Congenital Hip) OR (Displacement, Congenital Hip) OR (Dysplasia, Congenital Hip) OR (Hip Displacement, Congenital) OR (Congenital Hip Displacements) OR (Displacements, Congenital Hip) OR (Hip Displacements, Congenital) OR (Hip Dysplasia, Congenital) OR (Congenital Dysplasia Of The Hip) OR (Dislocation Of Hip, Congenital) OR (Hip Dysplasia, Congenital, Nonsyndromic) #2: “Ultrasonography”[MeSH] OR (Echotomography) OR (Diagnostic Ultrasound) OR (Diagnostic Ultrasounds) OR (Ultrasound, Diagnostic) OR (Ultrasounds, Diagnostic) OR (Sonography, Medical) OR (Medical Sonography) OR (Ultrasound Imaging) OR (Imaging, Ultrasound) OR (Imagings, Ultrasound) OR (Ultrasound Imagings) OR (Echography) OR (Ultrasonic Imaging) OR (Imaging, Ultrasonic) OR (Echotomography, Computer) OR (Computer Echotomography) OR (Tomography, Ultrasonic) OR (Ultrasonic Tomography) OR (Diagnosis, Ultrasonic) OR (Diagnoses, Ultrasonic) OR (Ultrasonic Diagnoses) OR (Ultrasonic Diagnosis) #3: “Graf” #4: #1 AND #2 AND #3
EMBASE (OvidSP)	#1: congenital hip dislocation/exp #2: “echography”/exp #3: “Graf” #4: #1 AND #2 AND #3
LILACS	#1: mh: “Luxação Congênita de Quadril” OR (Luxación Congénita de la Cadera) OR (Hip Dislocation, Congenital) OR (Congenital Dysplasia Of The Hip) OR (Congenital Hip Dislocation) OR (Congenital Hip Dislocations) OR (Congenital Hip Displacement) OR (Congenital Hip Displacements) OR (Congenital Hip Dysplasia) OR (Congenital Hip Dysplasias) OR (Dislocation Of Hip, Congenital) OR (Dislocation, Congenital Hip) OR (Dislocations, Congenital Hip) OR (Displacement, Congenital Hip) OR (Displacements, Congenital Hip) OR (Dysplasia, Congenital Hip) OR (Dysplasias, Congenital Hip) OR (Hip Dislocations, Congenital) OR (Hip Displacement, Congenital) OR (Hip Displacements, Congenital) OR (Hip Dysplasia, Congenital) OR (Hip Dysplasia, Congenital, Nonsyndromic) OR (Hip Dysplasias, Congenital) OR (Hip, Dislocation Of, Congenital) OR mh:C05.660.449 OR mh:C16.131.621.449 #2: mh: “Ultrassonografia” OR (Ultrasonografía) OR (Ultrasonography) OR (Ecografia) OR (Ecotomografia Computador) OR (Sonografia Médica) OR (Ecografia Médica) OR (Tomografia Ultrassônica) OR (Diagnóstico Ultrassom) OR (Imagem Ultrassônica) OR (Imagem Ultrassonográfica) OR (Imagem Ultrassom) OR (Imagem Ultrassom) OR (Ecotomografia) OR mh:E01.370.350.850$ #3: “Graf” #4: #1 AND #2 AND #3
CINAHL	#1: Hip Dislocation, Congenital #2: Ultrasonography or ultrasound or sonography or echography #3: Graf #4: #1 and #2 and #3
